# Dataset of solubility values for organic compounds in binary mixtures of solvents at various temperatures

**DOI:** 10.1038/s41597-026-07047-z

**Published:** 2026-03-20

**Authors:** Dmitry Malikov, Lev Krasnov, Marina Kiseleva, Elizaveta Meshcheriakova, Fedor Kuznetsov, Vladimir Elistratov, Matvei Vasiyarov, Sergei Tatarin, Stanislav Bezzubov

**Affiliations:** 1 N.S. Kurnakov Institute of General and Inorganic Chemistry, Russian Academy of Sciences, 119991 Leninskii pr. 31, Moscow, Russia; 2 Department of Chemistry, M.V. Lomonosov Moscow State University, 119991 Moscow, Lenin’s Hills 1/3, Russia; 3 National Research University Higher School of Economics, 7 Vavilova Str., Moscow, 117312 Russia

## Abstract

Solubility is a crucial property of organic compounds, impacting their potential applications in synthetic chemistry, materials science and drug design. Moreover, in technological processes mixtures of solvents are often utilized, making the solubility assessment more complicated. Predicting solubility values in mixtures of solvents from a molecular structure can help to address this issue, although a large and diverse dataset is needed to effectively pursue data-driven studies. In this research, we present a dataset containing 175166 experimental solubility values within the temperature range from 252 to 383 K for 810 organic compounds possessing 3001 unique solute-binary solvent systems and 750 unique binary solvent mixtures extracted from 1115 peer-reviewed articles. The solubility data and molecular structures of solutes and solvents are translated to a unified machine-readable format, facilitating data analysis and machine learning model development. An interactive online tool for visualization and navigation through the data has also been developed. This dataset can serve as a comprehensive benchmark for predicting solubility in mixtures of solvents.

## Background & Summary

Solubility, being defined as a maximal possible concentration of a solute under certain conditions (temperature, pressure, solvent), is a very important property for compounds. Not only does it affect their synthesizability and guides optimization of reaction conditions^1–3^, but also defines the possibility of crystallization^4,5^, design of polymers^6^ and remains a cornerstone in the drug design process. In the latter case, the desired parameter is the aqueous solubility, and it is important at every stage - from cytotoxicity measurements to pharmacokinetic studies^7^ and finally to the development of bioavailable forms^8^. The use of solvent mixtures can synergistically improve the solubility of a certain compound^9^, giving access to a more diverse set of reaction media and crystallization conditions. Still, accurate measurement of solubility is a complicated, time- and labor-consuming process, especially for high-throughput scale studies usually required in process optimization or drug design pipeline. Determination of solubility in solvent mixtures becomes even more complicated as it also depends on the ratio between the cosolvents. Thus, development of predictive tools to estimate molecular solubility is still an important challenge in modern cheminformatics.

Several methods were already applied to pursue this task, including molecular dynamics^10,11^ or ab initio calculations^12,13^. Modern machine learning has emerged as one of the most promising approaches for molecular properties (in particular, solubility) prediction^14,15^, given its data-driven nature and ultimate potential for tunability. From the first and second solubility challenges initiated by Llinas *et al*.^16,17^, numerous attempts were executed to develop machine learning and deep learning models towards accurate and generalizable solubility prediction, resulting in the development of a data-driven platform for solvent selection^18^. To achieve desirable accuracy and generalizability, researchers highlighted the strong need for large high-quality solubility datasets^19,20^. For the aqueous solubility, as it is involved in health-related applications and thus is the most popular to predict, AqSolDB still remains the most popular dataset^21,22^, yet the in-house data have also been used in some recent studies^23^. Prediction of solubility in organic solvents remained less explored, though rational selection of descriptors and curation/usage of corresponding datasets helped to overcome this obstacle^24–26^. However, release of BigSolDB^27^ and further update as BigSolDB 2.0^28^ also gave researchers access to a large amount of high-quality data, followed by a series of studies, which included the use of graph neural networks with specific solvent-solute interactions^29–31^, classical gradient boosting with molecular descriptors^19,32,33^, ensemble learning models^34^ or fastprop/chemprop architectures^35,36^. It should be noted that both quantity and quality of solubility data are important^20,29^ yet not exclusive^37^ conditions for successful machine learning models development.

As for the prediction of solubility in solvent mixtures, Zhang *et al*. trained a Jouyban-Acree-Artificial Neural Network on a proprietary dataset curated from Reaxys^38^. Bao *et al*. pioneered in the field of creating public datasets^39^, compiling c.a. 27000 solubility datapoints, developing machine learning models (MAE = 0.33 on test set for LightGBM) and validating them on certain experimental entries. However, for example, prediction of celecoxib solubility was relatively inaccurate, which was attributed to a deviation of its main features from the dataset distribution, implicitly suggesting need for enlargement of the dataset. Kim *et al*.^40^ leveraged graph neural networks (GNNs) for accurate prediction of dGsolv in solvent mixtures, but the part of the dataset comprising binary and ternary mixtures was only slightly enlarged, resulting in total of 31000 datapoints. Vermeire *et al*.^41^ also aimed at thermodynamic characteristics of the solubility process and used Dortmund Data Bank and DIPPR database to calculate a dataset of 34272 dGsolv, which was further used to develop a novel SolProp-mix GNN architecture. Nevertheless, in these works as well as in some other studies^42,43^ limited diversity and scale of the datasets are still observed.

In this work, we aimed at curation of the comprehensive experimental solubility dataset of organic compounds in binary mixtures of solvents, resulting in total of 175166 experimental solubility values within the temperature range from 252 to 383 K for 810 organic compounds possessing 3001 unique solute-binary solvent systems and 750 unique binary solvent mixtures extracted from 1115 peer-reviewed articles. This dataset provides a benchmark for the development of novel machine learning and deep learning models for predicting solubility in mixtures of solvents.

## Methods

**Fig. 1:**
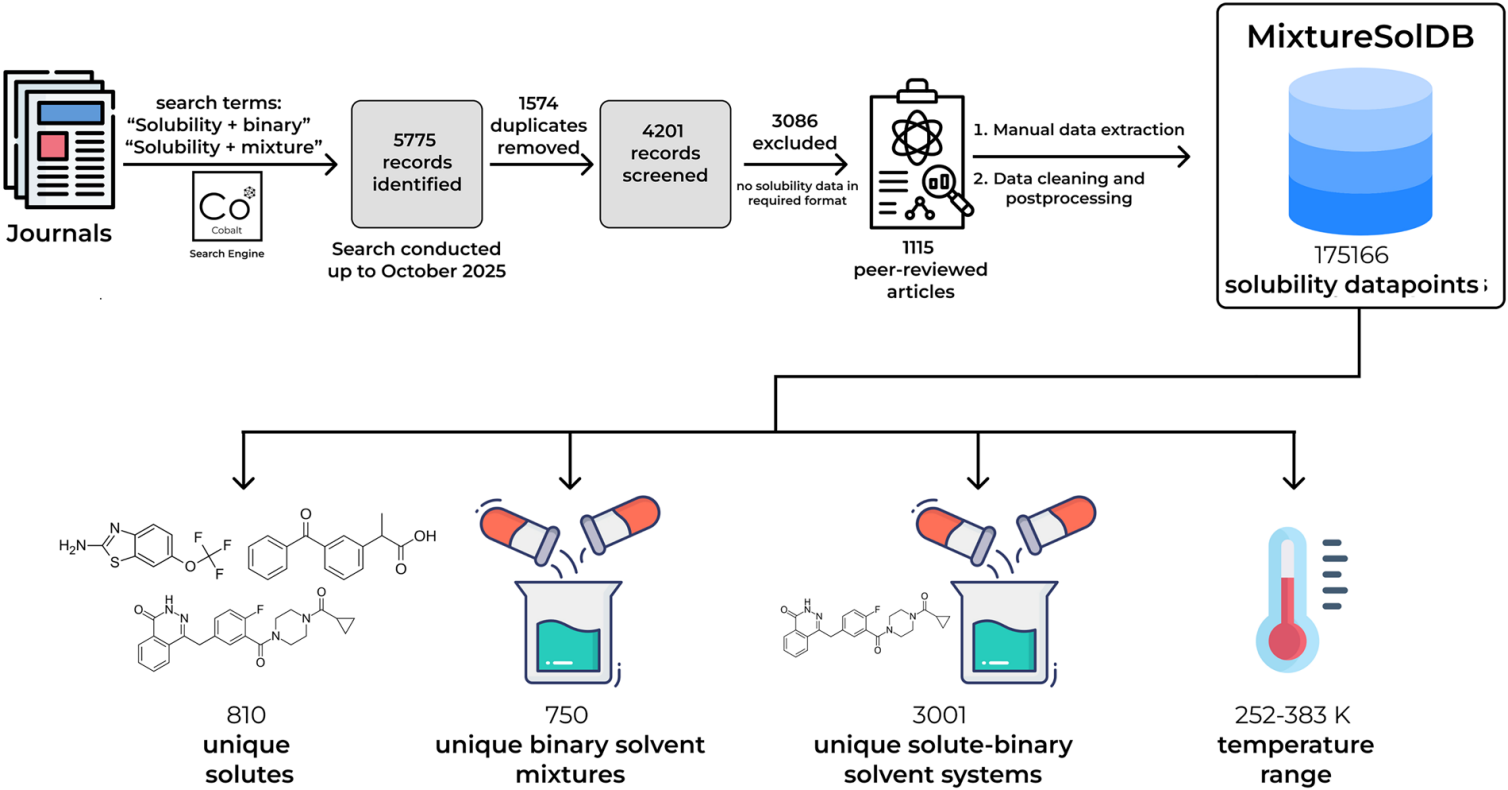
The general scheme of MixtureSolDB collection.

The scheme of MixtureSolDB construction significantly resembles the creation of BigSolDB 2.0^28^. The general scheme of the data curation is presented in Fig. 1. First, from the peer-reviewed journals, using freely available Cobalt search engine (the example of an exact query string is as follows: https://cobalt.colab.ws/FWqUQkX6), articles containing the word combinations “solubility + binary” or “solubility + mixture” in the title and/or abstract were extracted. The vast majority of such articles were only from several journals (Journal of Molecular Liquids, Journal of Solution Chemistry, Journal of Chemical & Engineering Data, Chinese Journal of Chemical Engineering, Physics and Chemistry of Liquids and Fluid Phase Equilibria), which aligns well with their scopes. Our search algorithm obviously imposes restrictions on the language - only articles in English were considered. The literature search was conducted up to October 2025. A total of 5775 records were identified using two query combinations (“solubility + binary” and “solubility + mixture”), of which 1574 duplicates were removed, leaving 4201 unique records screened. Of these, 3086 were excluded as they contained no usable solubility data in the required format, and 1115 articles met the inclusion criteria and were subjected to manual data extraction, what is reflected on a PRISMA-style diagram presented on Fig. 1. The articles chosen for data extraction contained molar solubility values expressed as mole fraction and cosolvent fraction values expressed as either mole fraction or mass fraction. Regarding the composition of the dataset, all organic compounds, organic salts (with either organic cation and/or anion) and solvates (with defined composition) were included. If a certain polymorphic modification was studied (implying existence of several modifications), it was included only if in the source article was a direct statement that it is the most stable one. Only measurements under atmospheric pressure and without any extra additives were added to the database. All molecular structures were converted to SMILES format using PubChem (https://pubchem.ncbi.nlm.nih.gov/). If the solute molecule was not presented in the PubChem database, the corresponding SMILES was generated by ChemDraw 18.0. The SMILES were canonized using RDKit (https://www.rdkit.org). This was followed by cleaning and post-processing of the data.

The processing of data included:

- Unification of solvent notations in order to eliminate ambiguity. For example, “1-propanol”, “propanol”, and “propan-1-ol” were converted to “n-propanol”. When the solvent names were unified, their SMILES representations were generated using PubChem. For abbreviations, we have also provided a supplementary table with IUPAC name for the each abbreviation, it is presented on Zenodo with the main dataset.
- Conversion to LogS units. The most commonly used data representation for data-driven solubility prediction is LogS, where S stands for molar solubility, owing to its more uniform representation. In this research we converted all the solubility values to LogS(g/100g) values for all the solvent mixtures, including those with PEGylated solvents (for these molecular weights were assigned based on the specific PEG grade reported in the source article, using their nominal average molecular weights for the conversion), in order to ensure consistency with the previous databases^19,39^. For this purpose, molecular weights of solvents and solutes were automatically calculated with RDKit and the conversion was executed. The formulas for conversion of S(molar fraction) to S(g/100g) are as follows: ${S}_{{\rm{g}}/100{\rm{g}}}=\frac{\left(\frac{x}{1-x}\right){M}_{{\rm{solute}}}}{w\,{M}_{{\rm{solvent1}}}+(1-w)\,{M}_{{\rm{solvent2}}}}\times 100$, if the fraction of Solvent1 is expressed as a mole fraction, where *M*_*solute*_, *M*_*solvent*1_ and *M*_*solvent*2_ correspond to molecular masses of solute and both solvents, x corresponds to mole fraction and w stands for the mole fraction of Solvent1 in the solvents mixture. ${S}_{{\rm{g}}/100{\rm{g}}}=\left(\frac{x}{1-x}\right){M}_{{\rm{solute}}}\left(\frac{w}{{M}_{{\rm{solvent1}}}}+\frac{1-w}{{M}_{{\rm{solvent2}}}}\right)\times 100$, if the fraction of Solvent1 is expressed as a mass fraction, where *M*_*solute*_, *M*_*solv*1_ and *M*_*solv*2_ correspond to molecular masses of solute and both solvents, x corresponds to mole fraction and w stands for the mass fraction of Solvent1 in the solvents mixture.
- Including additional information about the solutes. Namely, PubChem API(https://github.com/mcs07/PubChemPy) was used to extract CAS numbers, names as well as PubChem CID. ChEMBL web resource client (https://github.com/chembl/chembl_webresource_client) was used to extract the information whether the solute is an FDA-approved drug. It should be noted that there are several solutes, for which we opted for trivial names instead of IUPAC names, as these are commonly used.

## Data Records

MixtureSolDB can be accessed online within Zenodo^44^. The main dataset is structured as a downloadable CSV format data record. Each row of the dataset corresponds to an experimentally reported solubility measurement of a solute in a binary solvent system of defined composition at a given temperature. It should be noted though, that 28,870 entries, or about 16.5% of the dataset, correspond to solubility in pure solvents (marked with fractions of 0 or 1, respectively), enabling seamless comparison and analysis of these data to the solubilities in solvent mixtures. Description of each available metadata field as well as the potential range/constraints are provided in Table 1. The additional dataset of solvents boiling points is also provided as a downloadable CSV format data record, its description is provided in Table 2.

**Table 1:** Description of each metadata field for the main dataset.

Column	Description	Type	Range / constraints
RecordID	stable unique identifier for each dataset row	string	unique
SMILES_Solute	SMILES representation of the solute molecule	string	valid SMILES
Temperature_K	temperature for the reported solubility value, K	float	typically 250-400 K
Solubility(mole_fraction)	the reported solubility value expressed as mole fraction of solute	float	0 < x < 1
LogS(mole_fraction)	decimal logarithm of the solubility expressed as mole fraction of solute	float	unbounded
Solubility(g/100g)	the recalculated solubility value expressed as grams of solute per 100 g of solvent	float	>0
LogS(g/100g)	decimal logarithm of the solubility expressed as grams of solute per 100 g of solvent	float	unbounded
Solvent1	name of the first solvent component in the solvent mixture	string	non-empty string
Solvent2	name of the second solvent component in the solvent mixture	string	non-empty string
SMILES_Solvent1	SMILES representation of the first solvent component	string	valid SMILES
SMILES_Solvent2	SMILES representation of the second solvent component	string	valid SMILES
Fraction_Solvent1	initial fraction of the first solvent component in the solvent mixture (before solute addition), expressed according to Fraction_Type	float	[0,1]
Fraction_Solvent2	initial fraction of the second solvent component in the solvent mixture (before solute addition), expressed according to Fraction_Type	float	[0,1]
Fraction_Type	fraction type for Fraction_Solvent1 and Fraction_Solvent2	string	mole, mass
Compound_Name	solute name	string	non-empty string
CAS	solute CAS number	string	valid CAS or empty
PubChem_CID	solute PubChem CID	integer	positive integer or empty
FDA_Approved	indicates whether the solute is approved by the U.S. Food and Drug Administration (FDA)	boolean	True or False
Source	DOI of a data source for given values	string	valid DOI
IsPureSolventEndpoint	flag indicating whether the solvent mixture corresponds to a pure-solvent endpoint (Fraction_Solvent1 = 0 or 1)	boolean	True / False

**Table 2:** Description of each metadata field for the solvents boiling point dataset.

Column	Description	Type
Solvent	solvent name	string
Boiling_point_K	the reported boiling point value	float
Source	data source for given values	string

Each entry contains a stable ID in the format MSDXXXXXXX (e.g. MSD0175166). The solute molecule is represented as a SMILES string in the column SMILES_Solute. The experimental temperature of the solubility measurement (expressed in Kelvin) is reported in column Temperature_K. Solubility values are provided in several complementary representations. Solubility(mole_fraction) reports the experimentally measured solubility expressed as the mole fraction of the solute in the solvent system, while LogS(mole_fraction) gives the decimal logarithm of this value. The solubility value is also reported as grams of solute per 100 g of solvent (Solubility(g/100g)) and LogS(g/100g), respectively.

The names of the first and second components of the solvent mixture are given in Solvent1 and Solvent2 columns, while their corresponding SMILES representations are provided in SMILES_Solvent1 and SMILES_Solvent2 columns. The initial composition of the solvent mixture before solute addition, expressed as the fraction of the first solvent component, is reported in the column Fraction_Solvent1. To ensure unambiguous interpretation, solvent pairs were canonicalized by ordering solvents alphabetically: Solvent1 is always alphabetically ≤Solvent2. If necessary, the solvent fractions were transformed accordingly. The column Fraction_Type indicates whether the solvent fraction is expressed as a mole fraction or a mass fraction. Also, Fraction_Solvent2 was added as a separate column as well as the flag IsPureSolventEndpoint to easily detect the entries corresponding to pure solvents.

Additional metadata identifying the solute are provided in Compound_Name, CAS, and PubChem_CID columns, which contain the solute name, CAS registry number, and PubChem compound identifier, respectively. In the dataset 130 entries are left with blank CAS and 522 entries are left with blank PubChem_CID. For these entries, IDs describing the exact structural composition do not exist in the corresponding databases. The column FDA_Approved indicates whether the solute is approved as a drug by the U.S. Food and Drug Administration, with approved compounds labeled as “True” and all others labeled as “False”. The Source column provides the DOI of the original literature reference from which the corresponding solubility data were extracted.

## Data Overview

**Fig. 2:**
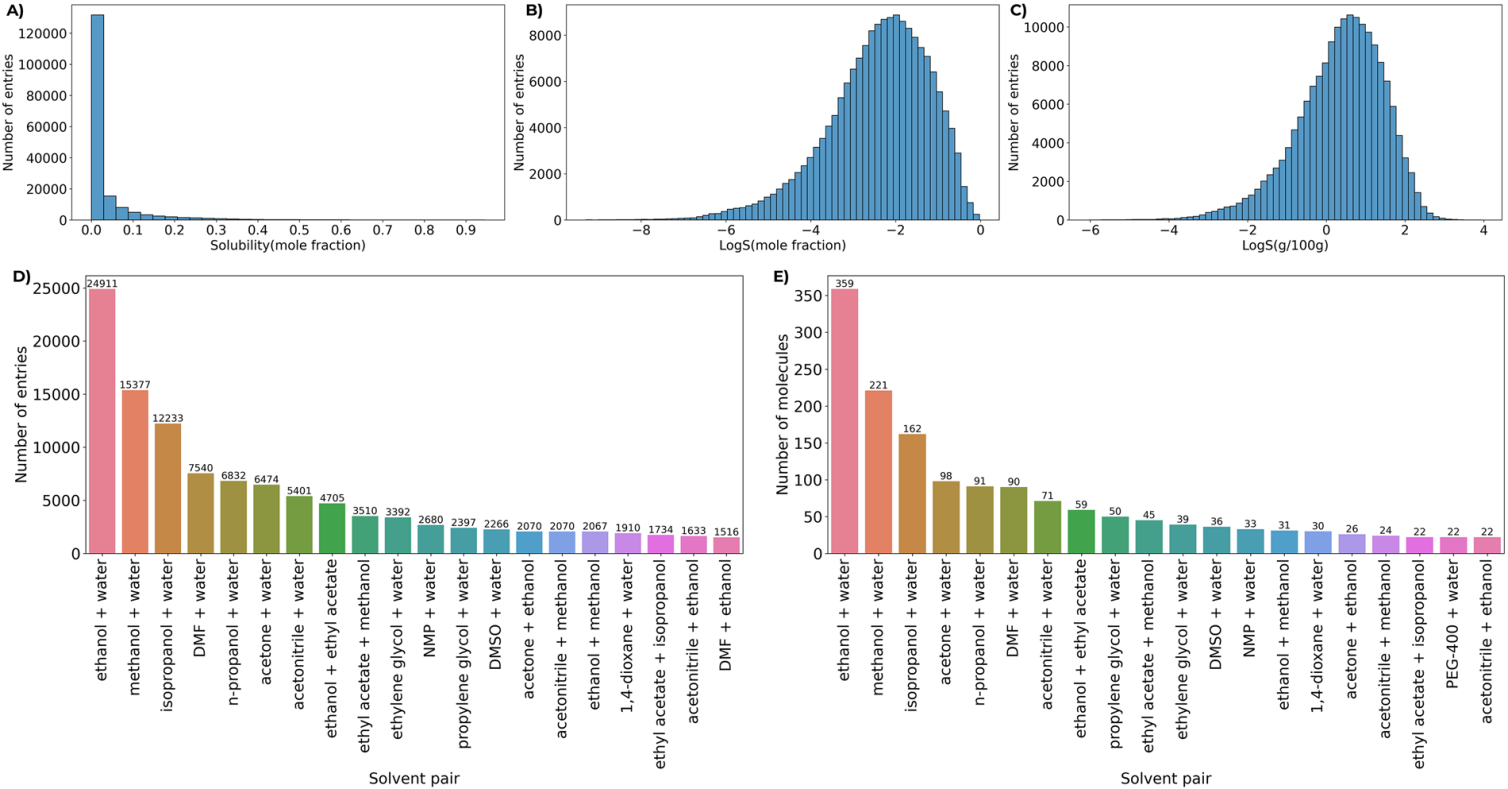
Statistics in the MixtureSolDB. (**A**) Distribution of entries based on mole fraction solubility values. (**B**) Distribution of entries based on LogS(mole fraction) solubility values. (**C**) Distribution of entries based on LogS(g/100g) solubility values. (**D**) Distribution of the solvent pairs by number of entries (top 20 solvent pairs are presented). (**E**) Distribution of the solvent pairs by number of unique solutes (top 20 solvent pairs are presented).

The detailed statistics of the dataset are presented in Fig. 2. One can notice that the vast majority of molecules display relatively low absolute solubility values with mole fraction <0.01 (104635 entries, 60%), so such distribution is not fully representative (Fig. 2A). However, the logarithmic representation is more universal and shows a distribution that is closer to normal, with a maximum around LogS(mole fraction) = −2 and extended low- and high-solubility tails, with approximately 10% of the data below LogS  = −4 and 10% above LogS  = −1 (Fig. 2B,C). The most common solvent pairs, both in terms of the number of experimental entries and the number of unique solute molecules, always include water, mixing with low-molecular-weight alcohols, ethyl acetate, dimethylformamide, acetone, or acetonitrile (Fig. 2D,E). Overall, water is one of the solvent components in 57% of solvent pair entries.

In comparison with BaoDB^39^ and MixSolDB^40^, the MixtureSolDB dataset has been significantly expanded. Although being still noticeably biased to water-containing systems, it encompasses a substantially larger number of solubility datapoints, solutes, binary solvent mixtures and unique solute-binary solvent systems leading to a broader coverage of chemical space, as summarized in Table 3.

**Table 3:** Comparison of MixtureSolDB dataset with the previous publicly available datasets.

	BaoDB (2024)^39^	MixSolDB (2025)^40^	MixtureSolDB (This work)
Solubility datapoints	28098	34734	175166
Solutes	125	171	810
Solvents	44	95	139
Binary solvent mixtures	110	153	750
Solute-binary solvent systems	373	545	3001
Sources	126	209	1115

## Technical Validation

The data in the current research were extracted from scientific articles, which were already peer-reviewed. Thus, the main source of errors in our dataset can be related to mistakes during data extraction. In order to maintain validity and consistency of the dataset, we performed cross-checking of the data, adapted from our previous studies^28,45^. Two people with at least graduate-level education in physical chemistry separately collected the solubility data from the papers. The third person compared these two datasets and merged them to the final dataset. The inconsistencies were double-checked by going back to the source article, and the correct variant was added. After that the resulting dataset underwent additional rechecking to ensure completeness and clarity of the data. The process of rechecking the dataset included the following steps:

- We have checked and fixed all entries with erroneous mole fraction values of solutes exceeding 1.
- We have checked and fixed all entries with mole or mass fractions of Solvent1/Solvent2 exceeding 1.
- We have additionally reexamined all the entries, where the measurement temperature lay near (10 degrees below/above) one of the solvents boiling point (see Table 2 for details on the boiling points subset).
- We have checked the validity of all sources by checking all DOIs using CrossRef API(https://github.com/CrossRef/rest-api-doc)
- We have checked cases in which one unique name corresponded to two different SMILES and vice versa. This was done to eliminate potentially incorrect ‘Compound_Name’ and ‘SMILES_Solute’ designations. In all the abovementioned cases we have gone back to the original data source and navigated through the original data in order to eliminate any potential confusions.

## Usage Notes

The data was compiled to propose a benchmark dataset allowing one to:

- perform straightforward analysis of a large amount of solubility data in solvent mixtures. For this purpose an online interactive tool, allowing such analysis without the need to process raw data, was developed: https://mixturesoldb.streamlit.app/. Within the website it is possible to search solutes by compound name, to search solutes by chemical structure as well as to filter only FDA approved molecules. The data for a certain solute is presented both as 3D visualization and in tabular format.
- perform training of machine learning and deep learning models aiming to predict solubility in a wide variety of organic solvent mixtures for diverse arrays of organic compounds. It should be noted that for benchmarking purposes we recommend using LogS expressed in mole fraction, as it provides a dimensionless and cross-solvent comparable target, while mass-based solubility (LogS(g/100 g)) is more usable for practical relevance^39^, also being a proper target for ML models development.

## Data Availability

The code used in this study is available at https://github.com/levakrasnovs/MixtureSolDB/tree/main.
